# Weaknesses and capacities affecting the Prehospital emergency care for victims of road traffic incidents in the greater Kampala metropolitan area: a cross-sectional study

**DOI:** 10.1186/s12873-017-0137-2

**Published:** 2017-10-03

**Authors:** Joseph Kimuli Balikuddembe, Ali Ardalan, Davoud Khorasani-Zavareh, Amir Nejati, Owais Raza

**Affiliations:** 1 Department of Disaster Public Health, School of Public Health, Tehran University of Medical Sciences, Tehran, Iran; 2 Tehran University of Medical Sciences–International Campus, Tehran, Iran; 3 East African Center for Disaster Health and Humanitarian Research, Kampala, Uganda; 4 National Institute of Health Research, Tehran University of Medical Sciences, Tehran, Iran; 5 Harvard Humanitarian Initiative, Harvard University, Cambridge, USA; 6 Safety Promotion and Injury Prevention Research Center, Shahid Beheshti University of Medical Sciences, Tehran, Iran; 7 Department of Health in Disaster and Emergency, School of Health, Safety and Environment, Shahid Beheshti University of Medical Sciences, Tehran, Iran; 8 Post-Doc Research Fellowship, Department of Clinical Science and Education, Karolinska Institute, Stockholm, Sweden; 9 Department of Emergency Medicine, Imam Khomeini Hospital, Tehran University of Medical Sciences, Tehran, Iran; 10 Department of Epidemiology and Biostatistics, School of Public Health, Tehran University of Medical Sciences, Tehran, Iran

**Keywords:** Uganda, Kampala, Road traffic incidents, Prehospital, Emergency medical services

## Abstract

**Background:**

Pre-hospital emergency care is a vital and integral component of health systems particularly in the resource constrained countries like Uganda. It can help to minimize deaths, injuries, morbidities, disabilities and trauma caused by the road traffic incidents (RTIs). This study identifies the weaknesses and capacities affecting the pre-hospital emergency care for the victims of RTIs in the Greater Kampala Metropolitan Area (GKMA).

**Methods:**

A cross-sectional study was conducted in the GKMA using a three-part structured questionnaire. Data related to the demographics, nature of RTIs and victims’ pre-hospital experience and existing Emergency Medical Services (EMS) were collected from victims and EMS specialists in 3 hospitals and 5 EMS institutions respectively. Data was descriptively analyzed, and after the principal component analysis was employed to identify the most influential weaknesses and capacities affecting the pre-hospital emergency care for the victims of RTI in the GKMA.

**Results:**

From 459 RTI victims (74.7% males and 25.3% females) and 23 EMS specialists (91.3% males and 8.7% females) who participated in the study between May and June 2016, 4 and 5 key weaknesses and capacities respectively were identified to affect the pre-hospital emergency care for RTI victims in the GKMA. Although some strengths exist like ambulance facilitation, EMS structuring, coordination and others), the key weaknesses affecting the pre-hospital care for victims were noted to relate to absence of predefined EMS systems particularly in the GKMA and Uganda as a whole. They were identified to involve poor quality first aid treatment; insufficient skills/training of the first responders; inadequate EMS resources; and avoidable delays to respond and transport RTI victims to medical facilities.

**Conclusions:**

Though some strengths exist, the weaknesses affecting prehospital care for RTI victims primarily emanate from the absence of predefined and well-organized EMS systems in the GKMA and Uganda as a whole.

**Electronic supplementary material:**

The online version of this article (10.1186/s12873-017-0137-2) contains supplementary material, which is available to authorized users.

## Background

Road traffic incidents (RTIs) are responsible for the largest proportion of unintentional injury and mortalities in the world. At present, they are estimated to be the ninth leading cause of deaths globally at 1.25 million each year especially among those aged 15–44 years. They are predicted to become the 5th leading cause of deaths by 2030 unless urgent actions are taken [1, 2]. However, a disproportionate burden of RTIs is borne mostly by the low and middle-income countries (LMICs). The LMICs which are much less motorized, experience 85% of the global RTIs compared to the high income countries (HICs) with 15% and yet they are largely motorized [1, 3, 4]. Considerable socioeconomic costs result from the deaths, injuries and serious disabilities due to RTIs. This places a considerable burden on the public health systems. As if that is not bad enough, RTIs also bring serious economic impacts as the lives lost often represent a loss of physical manpower, skills, experience and knowledge that is not easy to replace. In the LMICs the economic loss due to RTIs is estimated to cost countries between 1 and 3% of their total Gross Domestic Product (GDP) per annum [1–3].

Uganda, being one of the LMICs, is no exception, with a disproportionately high number of RTIs reported each year [5–7]. It was recently reported that 27.4 per 100,000 of the population die annually in RTIs in Uganda [1, 6, 8, 9]. This exceeds the global average rate of 17.4 per 100,000 population according to World Health Organization (WHO) [1]. The majority of all the registered RTIs happen in the Greater Kampala Metropolitan Area (GKMA) more than anywhere else in Uganda [6, 10]. In response, concerted road safety strategies covering a variety of approaches have been put in place to respond to this worryingly high rate of RTIs particularly in the GKMA and Uganda in general. But much more is still needed to be done to improve post-crash management and the ability of the health systems to provide appropriate emergency treatment for crash victims as called for by the Decade of Action for Road Safety (2011–2020) [11]. However, that should be attuned as much as possible to the needs of victims and existing human, finance and equipment resources. This paper will now venture to comprehensively explore the weakness and capacities which affect the pre-hospital emergency care for victims of RTIs in the GKMA.

## Methods

### Design

A cross-sectional study was conducted to identify the weaknesses and capacities affecting the pre-hospital emergency care for the victims of RTIs in the GKMA. In a cross-sectional method either the entire population or a subset thereof is selected, and from this, data is collected to help answer research questions of interest. Besides other considerations, in recognizing the importance and trend of RTIs in the GKMA in particular, we choose to employ the cross-sectional design as an effective method to select a desired proportion of respondents as representatives of the RTI victims as well as the EMS specialists who attend to those affected.

### Study setting

This study was conducted in the GKMA, which covers a radius of 40 Km^2^ (1000 Km^2^ total area) from Kampala capital city to the nearby cities of Entebbe, Kira, Mpigi, Mukono, Nansana and Wakiso–which is at times referred to as the Kampala Metropolitan Extra region (KMA) [12–14]. The GKMA is the fastest developing region in Uganda due to its close vicinity to Kampala city. Its current population is estimated at ~3.5 million, with an annual growth rate of 5% per annum [8, 14]. Kampala and the GKMA connect the country’s traffic, and act as the major business and industrial hub of Uganda, contributing to over 70% of the country’s industrial production and over 60% of the country’s GDP [12]. Due to this status, the GKMA is confronted with various challenges which include the abnormally high burden of RTIs. Available data indicates that roughly half of total RTIs; 12,152 (54%); 12,136 (54%) and 9651 (53%) occurred in the GKMA of the total crashes registered in Uganda; 22,461, 22,272 and 18,368 in 2010, 2011 and 2013 respectively in Uganda as a whole [5–7]. That continues to necessitate the urgent need for more road safety vis-à-vis appropriate pre-hospital EMS actions to bring the situation under control.

### Study participants and context

Two groups of RTI victims and EMS specialists (first emergency responders, paramedics, nurses and ambulance crew); 459 and 23 respectively were involved in the study for a period of 2 months–May and June, 2016. The former were recruited from three major hospitals: Mulago National Referral Hospital (MNRH), China- Uganda Friendship Hospital Naguru (CUFH-N) and Kibuli Muslim Hospital (KMH). It is worth stating that the majority of RTI victims and trauma causalities in Kampala and Uganda are presented mostly to the MNRH–the main and public national referral hospital whose services are meant to be offered free of charge. It is regularly presented with an abnormal number of patients with different illnesses. MNRH is operated as a teaching hospital at a tertiary level with a capacity of between 1200 and 1500 beds, and with a 24 h accident and casualty department [15]. A considerable number of RTI victims are presented to CUFH-N which is also a public general hospital that was established to relieve the MNRH. Other private hospitals within the GKMA like KMH are also presented with RTI victims. The EMS specialists were culled by the principal investigator from the three hospitals, and other five institutions offering EMS in the GKMA as indicated in Table 2. Given that this study is cross-sectional in nature, a combination of the two categories of respondents was considered to be representative.

### Participants’ inclusion

To be included in the study, a RTI victim mainly had to have been injured in the GKMA within the previous 30 days. This period was considered appropriate since it could easily enhance the data and information collection based on how the victims’ or guardians’ could recall their RTI experience. Also he/she had to be either an out-patient or in-patient seen to or admitted to the accident and emergency department of the MNRH, the CUFH-N or the KMH. Any RTI victims who were injured outside the GKMA and/or referred from other hospitals or medical facilities to any of the 3 hospitals were excluded. On the other hand, the EMS specialists who were selected to the study had to be well-informed about EMS and to possess work experience related to EMS of not less than 2 years.

### Sample size

Non-probabilistic convenient sampling was used given that a certain proportion of respondents was needed to be drawn from the study population. In this case, 459 victims of RTIs were recruited in the study from the overall numbers of victims presented in the 3 hospitals within the 2 months. The victims were distributed amongst the 3 hospitals as follows: 373 (81.2%); 54 (11.7%) and 32 (6.9%) for MNRH, CUFH-N and KMH respectively. The highest number was selected from the MNRH since it is the main referral hospital where the majority of injury and trauma patients are presented across the GKMA followed by the CUFH-N. We also decided to consider the KMH among other private hospitals. Similarly, purposive sampling was employed to select in the EMS specialists who met the study inclusion criteria in the GKMA. As a result, of the 30 EMS specialists who were contacted only 23 (76.6%) of the EMS specialists responded and this number was still considered to form a representative sample.

### Data collection

A three part structured questionnaire organized with both open and close-ended questions and instructions and a consent form was used to collect the data (Additional file 1). It was prepared without following any EMS standard. At first, the questionnaire was reviewed by study’s first four authors and then it was pilot-tested with 10 RTI victims and 5 EMS specialists. That led to the adoption of its final version which was presented in both English and Luganda, the common languages used in the GKMA. The questionnaire consisted of Part A, B and C with 10, 9 and 23 questions respectively. Part A applied to all the participants, and it aimed to capture their demographic data and information (age, gender, place of living, nationality, employment, work experience and others). Part B only applied to the victims, and sought their information on the nature of their RTI and the details of their pre-hospital experience (place of crash, mode of crash and time, response time, responders, transport, injuries and treatment). Similarly, Part C only applied to the EMS specialists and captured varying information about the existing EMS in the GKMA (the ambulance types, equipment in use, dispatch arrangements, transport and communication capabilities; the qualifications and the training of EMS crews; triage and treatment; time, season and number of RTIs attended to; ambulance service payment and challenges involved in using it).

Four (4) trained interviewers were recruited, inducted and advised about the study and the questionnaires before they were deployed to conduct face-to-face interviews with victims of RTIs at the MNRH, the CUFH-N and the KMH. The estimation time for the interview needed to not exceed 10 min. The principal investigator personally distributed the questionnaires to the EMS specialists so that they could complete them in their own time, and where necessary he made follow-ups with reminders.

In this study, a RTI is defined as a road traffic injury incurred as a result of a collision on a public road involving at least one moving vehicle [16], while EMS is defined as the care delivered in the first few hours after the onset of an acute medical or obstetric problem or the occurrence of an injury, including care delivered inside a fixed facility [17]. This study also refers to a weakness as a loophole, limitation or inadequacy, while the capacity is identified as strength or an achievement in rendering the pre-hospital emergency care for RTI victims in the GKMA.

### Ethical considerations and procedures

This study is part of an entire research project which was approved by the Tehran University of Medical Sciences, the Office of the Vice Chancellor for Research and the Uganda National Council for Science and Technology [18, 19]*.* Official approvals from the Research and Ethics Committee were also sought and granted from the MNRH, the CUFH-N and the KMH. Also before distributing the questionnaires to different EMS specialists, written permission was requested and granted from their superiors or supervisors. Each participant who agreed to participate in the research had to consent either by a signature or finger/thumb print but only after the study’s objectives and its anticipated benefits were clearly explained to them. The participants were also assured that their data and information would be kept confidential.

### Data management and analysis

After the data collection process, the data from two sets of questionnaires (for victims and EMS specialists) was entered into the IBM Statistical Package for the Social Sciences (SPSS) version 22 [20] for descriptive analysis. The datasets were cleaned by removing any incomplete or missing data and this resulted in 41 cases (8.2%) being ruled out. For each of the two datasets, identical methods of analysis were followed. First, descriptive statistics (age groups; gender; place of living; marital status; nationality; education; occupation; work experience; organization and position) were used to describe the data. At the second stage, the principal component analysis (PCA) was deployed to extract variables influencing weaknesses and capacities from the RTI victims and EMS datasets respectively.

PCA is a multivariate technique that analyzes a data table in which observations are described by several inter-correlated dependent variables. The relationship between the variables and corresponding components can further be explained by studying the contribution of variables. The data is organized in the form of an (n x p) matrix X, with n observations, and p variables. An orthogonal transformation is applied to X. This results in r (≤ p) whereby the new variables are linearly uncorrelated. These are the principal components (PCs) of the data, and they are ordered as follows. The first PC accounts for the most of the variability in the original data. The second PC accounts for maximum amount of the remaining variability in the data, which is subject to the constraint that is uncorrelated with (that is orthogonal to) the first PC [21, 22].

PCAs were undertaken using *R* packages. Pairwise correlation tests were applied in an effort to remove the highly correlated variables (Pearson’s correlations *r* > 0.80). None of the nine variables from the victims’ data had *r* > 0.80, and thus all the variables were included into the PCA, that is-the place, mode of RTI, time, responder, mode of transport, treatment received, type of treatment and type of injuries. From the EMS dataset, 8 variables had *r* > 0.80 and were therefore removed from further analysis leaving only 15 variables-the notification; communication; types of ambulances; number of ambulances; team qualifications; communication equipment; emergency contact; victim’s transportation; service payer; type of EMS; the season of RTI; common type of RTIs; responder training; EMS procedures and EMS challenges. After converting all the variables into ordinal categories, a correlation matrix was used for the PCA analysis as this standardizes the data and avoids potential bias resulting from the inclusion of data with different data ranges. Based on the PCA, we diagonalized the correlation matrix of nine variables (shown in Table 3) on the nature of pre-hospital care interventions for RTI victims. Similarly, we also diagonalized the correlation matrix of 15 variables (shown in Table 4) on the general characteristics about the existing EMS. All the variables were auto-scaled to mean zero and variance units. Using the criteria of Cattell and Jaspers (1967) [22], and with the help of a scree plot, only the components with variances greater than 1 will be retained.

The contribution of 9 and 15 variables related to the weaknesses and capacities respectively affecting the pre-hospital emergency care were weighed against the construction of the principal components (PCs) through the PCA pairwise correlation tests. This involved detecting the most predominant variable(s) among others which influence the PC score and vice versa. A variable’s contribution is the square of the correlation coefficient between the variable and PC, divided by the sum of the squared correlation between all variables and the PC. This can tell us whether there is some variable that contributes significantly to that dimension with respect to the others.

## Results

### Distribution of victims of RTIs

From Table 1, the majority of RTI victims were Ugandans (96.7%), aged between 18 and 35 years (58.6%) and mostly males (74.7%). Also, the victims were disproportionately involved in crashes across the GKMA, with the majority (27.7%) from the KMA while the Rubaga Municipality had the least number of victims (10.5%). Primary (40.7%) was the commonest level of education for victims. Table 1 also shows that the majority of the victims were employed (61.2%).

**Table 1:** Descriptive characteristics of the victims involved in road traffic incidents in the Greater Kampala Metropolitan Area between March and September 2016

Victims of RTIs (*n* = 459)	
	Frequency (Percent)
Gender	
Male	343 (74.7)
Female	116 (25.3)
Age groups	
< 18 years	67 (14.6)
18–35 years	269 (58.6)
36–55 years	94 (20.5)
> 55 years	29 (6.3)
Scene of RTI	
Central	89 (19.4)
Rubaga	48 (10.5)
Kawempe	83 (18.1)
Nakawa	57 (12.4)
Makindye	55 (12)
KMA Extra	127 (27.7)
Marital status	
Single	189 (41.2)
Married	244 (53.2)
Widow/Widower	22 (4.8)
Others	4 (0.9)
Nationality	
Ugandan*	444 (96.7)
Others	15 (3.3)
Education	
Illiterate	25 (5.4)
Primary	187 (40.7)
Secondary	147 (32.0)
Graduates	85 (18.5)
N/A (Toddlers)	8 (0.2)
Others	7 (1.5)
Years of work	
Have never worked	129 (28.1)
< 1	29 (6.3)
5–1 years	114 (24.8)
6–10 years	92 (20.0)
11–15 Years	34 (7.4)
16–20 years	19 (4.1)
> 20 years	40 (8.7)
Occupation status	
Unemployed	109 (23.7)
Employed	281 (61.2)
Students/Pupils	59 (12.9)
Toddlers	8 (1.7)
Unknown	2 (0.4)

*Abbreviations*: *n* number, *RTIs* road traffic incidents, *N/A* not applicable

*All the 23 EMS specialists were of Ugandan nationality

### Distribution of EMS specialists

Table 2 shows that the majority of EMS specialists (56.5%) were aged between 36 and 55 years living in the Central Municipality and the KMA Extra region; 30.4 and 21.7% respectively. They were employed from different governmental and private institutions, with the highest percentage (43.5%) from the Ministry of Health-Uganda National Ambulance Service (UNAS). Most of the EMS specialists (78.2%) were graduates. Nearly one half (34.8%) of them had more than 10 years of work experience in the EMS. The EMS workforce in our sample is mainly composed of the senior EMS administrators, ambulance drivers, fire-fighters and rescue officers.

**Table 2:** Descriptive characteristics of the emergency medical specialists who participated in the study between March and September 2016

EMS Specialist (*n* = 23)	
	Frequency (percent)
Gender	
Male	21 (91.3)
Female	2 (8.7)
Age groups	
18–35 years	10 (43.5)
36–55 years	13 (56.5)
Place of living	
Central Municipality	7 (30.4)
Rubaga Municipality	1 (4.3)
Kawempe Municipality	2 (8.7)
Nakawa Municipality	4 (17.4)
Makindye Municipality	3 (13.0)
KMA Extra	5 (21.7)
Others	1 (4.3)
Marital status	
Single	5 (21.7)
Married	17 (73.9)
Widow/Widower	1 (4.3)
Organization	
Ministry of Health-UNAS	10 (43.5)
Uganda Police Force- Fire Brigade	3 (13.0)
AAR-Uganda Limited	1 (4.3)
Kibuli Muslim Hospital	4 (17.4)
St. John’s Ambulance	1 (4.3)
Mulago Hospital	2 (8.7)
Naguru Hospital	1 (4.3)
Uganda Red Cross	1 (4.3)
Education	
Primary	1 (4.3)
Secondary	4 (17.4)
Graduate	18 (78.2)
Years of work	
1–5 years	6 (26.1)
6–10 years	6 (26.1)
11–15 years	8 (34.8)
16–20 years	3 (13.0)
Work position	
Senior EMS Administrator/Manager	6 (26.1)
Ambulance Driver	6 (26.1)
Ambulance Officer	3 (13.0)
Fire fighter and rescue officer	5 (21.7)
Emergency care specialist	3 (13.0)

*Abbreviations*: *n* number, *EMS* emergency medical services, *KMA* Kampala Metropolitan Area, *UNAS* Uganda National Ambulance Services, *AAR* African Air Rescue

### Weaknesses in the pre-hospital emergency care for RTI victims in the GKMA

**Fig. 1:**
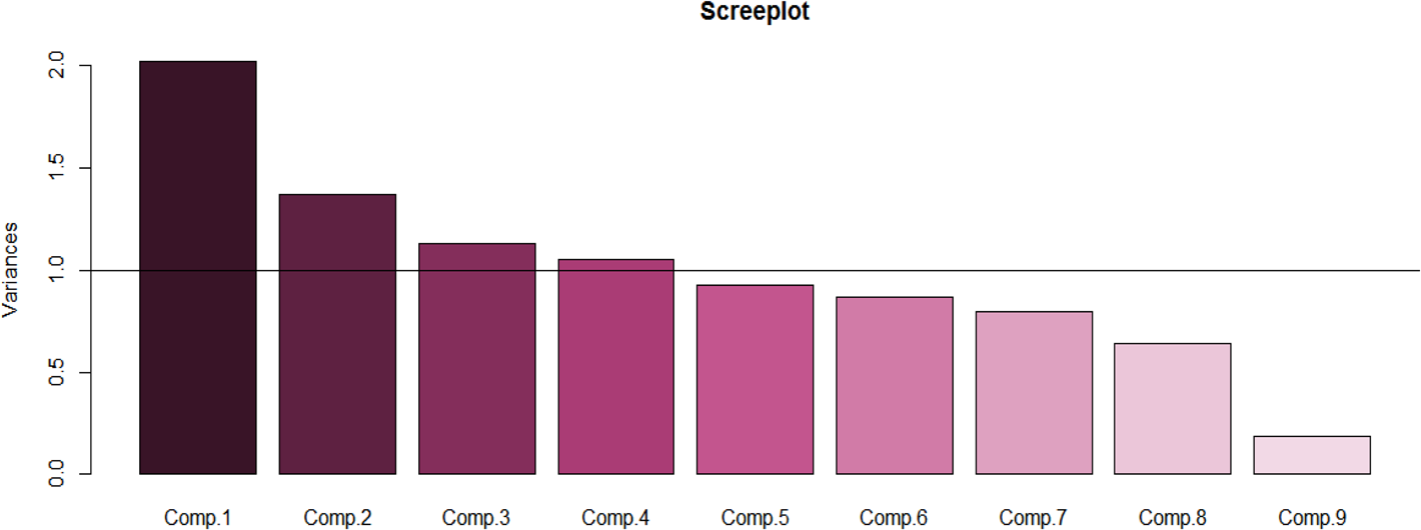
Scree plot showing 4 PC prehospital care weaknesses for RTI Victims in GKMA

Table 3 shows the PCA results on the loadings (correlated between components and the original variables), the eigenvalues for each component and percentage of variance (information) is explained by each component. These show the factors contributing to weaknesses in the pre-hospital emergency care for the victims of RTIs in the GKMA. In order to identify the principle components (PCs) to be retained, a scree plot was used as can be viewed by Fig. 1 below. By using this method, only the first four components with variances greater than 1 were retained, while PC5 and onwards were removed from further analysis. As also shown in Table 3, PC1, PC2, PC1, PC3 and PC4 are the components with the eigenvalues greater than one–these reflect components which contain more information than one original variable. The percentage of variance is the proportion of an eigenvalue for each component over the sum of eigenvalues.

**Table 3:** The retained principal components for weaknesses affecting the pre-hospital emergency care for victims of RTIs in the GKMA between March and September 2016

Variables	PC1	PC2	PC3	PC4
Location	−0.21	−0.12	0.64	0.10
Mode of RTI	0.14	−0.21	0.66	−0.20
Time of RTI	0.16	−0.17	−0.03	0.63
Provider of initial care	−0.29	0.48	0.02	0.38
Responder’s arrival time	0.21	−0.25	−0.23	0.25
Transportation of victim	−0.16	0.57	0.24	0.27
Treatment received by victim	0.61	0.29	0.15	0.00
Type of treatment	0.60	0.31	0.03	−0.08
Injuries of RTI victims	0.17	−0.34	0.14	0.52
Eigenvalues	2.02	1.37	1.13	1.05
Variance explained (%)	22.45	15.22	12.58	11.67
Overall weakness	Poor quality first aid treatment	Insufficient skills/ training of first responders	Inadequate EMS resources	Delays in timely response to victims

*Abbreviations*: *PC* principle component, *RTI* road traffic incident

As can be observed in Table 3, the first 4 PCs sum to 62% of the overall total variance from all the original variables. They were retained since their variable contributions were above 1 (Fig. 1). The first component (PC1) has the highest loadings (0.61 and 0.60) from the type and treatment received respectively. This can be considered to represent the poor first aid treatment which is given to the victims involved in RTIs. The victim’s transportation and provider of the initial care to the victim are highly correlated with PC2. This reveals the inappropriate skills and insufficient training of first responders in managing the treatment of victims both at the scene and in-transit to medical facilities. PC3 displays a high correlation with the mode and location of RTIs. This underscores the lack of, or inadequate distribution of EMS resources which basically related to facilities and personnel according to the locations of RTIs. In PC4, the time of RTIs and injuries sustained by the victim are strongly correlated, and this demonstrates the delay in the timely response in attending to the victims, which exacerbates the consequences of the injuries sustained by the victim.

Based on PCA pairwise correlation, the contribution of the first 2 most important variables within the 4 PCs were selected. In a scenario where all the variables have contributed equally, we would expect to see a balanced contribution from each variable. Thus, the variability in contribution reflects that not all the factors weigh equally in the component formation. For example, as can be observed in PC2-the transportation of victim variable is comparatively contributing more (32%) to the weakness “poor skills/training of responders” compared to the provider of initial care variable (23%). On the other hand, with PC4 the major contributors are the time of injury and injuries (40 and 27% respectively).

### Emergency medical service capacity in the pre-hospital emergency care in the GKMA

**Fig. 2:**
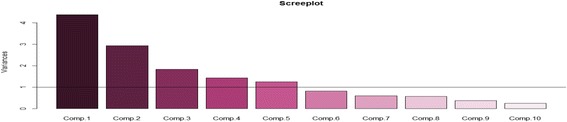
Scree plot showing 5 PC prehospital care capacities for RTI victims in GKMA

The PCA results obtained from the EMS specialist dataset are presented in Table 4 above. The first 5 PCs whose variable contributions were above 1 were retained and this is indicated by the scree plot (Fig. 2). They add-up to 79% of the total variance (Table 4). PC1 shows the highest loading with the type of EMS and the communication equipment. PC2 is largely correlated with the EMS team qualifications and emergency contacts. Also based on the PCA pairwise correlation test, the responders’ training and EMS challenges are highly correlated with PC3. With PC4, the highest loading was achieved by the common RTIs responded to and the communication between the responders. Lastly, PC5 is majorly influenced by the transportation of the victim and the EMS procedures followed.

**Table 4:** The 5 most important factors that enhance the capacities for pre-hospital emergency care for victims of road traffic incidents in the Greater Kampala Metropolitan Areas, March and September 2016

Variables	PC1	PC2	PC3	PC4	PC5
Notification	−0.29	−0.28	0.09	−0.29	−0.10
Communication between responders	−0.15	−0.17	0.33	−0.50	0.17
Ambulances used	−0.35	0.14	−0.29	−0.05	0.21
Number of ambulances	0.06	−0.41	−0.35	−0.06	−0.23
Team qualifications	−0.12	0.50	0.08	−0.07	−0.09
Communication equipment	−0.35	0.09	−0.34	−0.10	0.21
Emergency service center contacts	0.10	−0.45	−0.05	−0.22	0.17
Victim’s transportation	−0.29	−0.05	−0.30	0.17	−0.52
Service payer	−0.34	−0.00	0.10	0.12	0.23
Type of EMS	−0.41	0.14	−0.17	−0.00	0.20
RTI Seasons	−0.11	−0.39	−0.22	0.25	0.34
Most common RTIs responded to	−0.18	−0.20	0.18	0.58	−0.15
Responders’ training	−0.20	−0.11	0.47	0.25	0.19
EMS communication and transportation procedures	−0.27	−0.04	0.04	−0.29	−0.40
EMS challenges	0.32	0.12	−0.35	0.00	0.27
Eigenvalues	4.38	2.93	1.83	1.43	1.26
Variance explained (%)	29.18	19.57	12.23	9.55	8.39
Overall capacity/strength	Ambulance facilitation	Ongoing EMS structuring	EMS training	EMS coordination	Transportation procedures

*Abbreviations*: *PC* principle component, *EMS* emergency medical services, *RTIs* road traffic incidents

## Discussions

The findings of the present study indicate that the main weaknesses (the poor quality first-aid, insufficient skills or training of first responders, inadequate EMS resources and the delays in timely response to victims) identified to affect the pre-hospital systems are due to the absence of predefined and well-organized EMS systems particularly for the GKMA and Uganda as a whole. A number of studies have raised similar concerns particularly in the LMICs [23, 24], and some called for the urgent establishment of a definitive and well-planned emergency care system for Kampala and Uganda [10, 15, 25–27]. Despite the challenges there’s been some remarkable progress made in the pre-hospital emergency care for all hospital patients including those injured in RTIs in the GKMA and Uganda. The present study identified them to include: ambulance facilitation, ongoing EMS structuring, EMS training, EMS coordination and transportation procedures adhered to in the course of transporting the victims.

The provision of appropriate early emergency medical care at the RTI scene and on the way to hospital cannot only help to reduce the consequences of injuries but also prove critical in reducing mortality among the severely traumatized and injured victims [17, 28, 29]. This should be supported by the well-equipped EMS to enhance the first-aid treatment for RTI victims especially in the settings like the GKMA which are still constrained with limited EMS resources. However, based on the PCA assessment in this study the first-aid treatment for RTI victims in the GKMA is still far from adequate. This is often costing the lives of those severely injured in RTIs before they have even had a chance to receive definitive care at a medical facility. The potential reason of this is the insufficient skills among the first responders and the laypeople that arrive first at the RTI scene, a challenge this study has also identified. This is in-line with that of another study in Iran which also reported that untrained laypeople are often the first to arrive and gather at a crash scene [30]. The laypeople often consist of well-wishers, local community leaders, taxi drivers, Boda-bodas (commercial motorcyclists), bystanders, relatives and police. Therefore training of first responders and laypeople who frequently arrive at the scene of RTIs to attend to the victims can be effective in helping to minimize RTIs and their consequences as two studies in Ghana and Kampala-Uganda already proved [15, 25, 28, 31, 32].

In settings like the GKMA with no formal EMS system and lacking sufficient trained responders, the first tier of pre-hospital care may be composed of the laypeople who are taught the most basic first aid techniques [17]. Laypeople can play a significant role in rendering basic first-aid if they first undergo short courses for a few hours or days and practical orientations. The training should be didactic in nature and tailored to address the specific needs of their communities and the resources available. It should use local dialects to impart vital skills to the trainees on scene management, external compression for haemorrhage control, basic airway control, recovery position, safe lifting and transportation of injured victims, splinting fractures and triage [25, 28, 31, 33]. These skills can be applied to administer basic, but effective first-aid to those injured in the absence of any trained healthcare professionals immediately after the occurrence of a road crash. Evidence has shown that the first-aid trainees who were trained in any of the above skills had a positive impact on reducing mortality and decreasing trauma when they were followed-up. Accordingly, it was found that 97% of trainees in Kampala used at least one skill in the first 6 months after their basic first aid course while in Ghana, 61% of the 400 commercial taxi drivers who also underwent a basic first-aid course were reported to have utilized their skills to render first aid to injured patients [28, 34]. Also, the role of trained laypeople can also be enhanced by providing them with basic first-aid kits containing some simple equipment and supplies such as the gloves, gauze, cotton, bandage and others [28, 35, 36].

It should be noted that ordinarily RTIs are random events [30]. In this case, RTIs in the GKMA have been disproportionately occurring with some tending to be clustered in specific locations [6, 9]. With the challenge of nonexistent or inadequate EMS facilities and personnel which was found by the current study to hinder the pre-hospital care in the GKMA, this makes the provision and distribution of adequate EMS facilities and personnel a real challenge, especially in the low resource settings like the GKMA in particular and Uganda as a whole. To remedy this problem it makes sense to identify the areas-prone to high occurrences of RTIs and then allocate the scarce EMS resources-facilities and personnel accordingly and to target these areas with programs which mobilize and utilize the first-lay responders as has been discussed above. Other mechanisms to address the challenge of limited EMS resources can involve identifying the core functions of different stakeholders who can play a vital role in reducing RTIs. Adequate institutional capacity, funding, legal authority and human resources can then be better directed to support those core functions across the spectrum of injury control: surveillance, road safety and trauma care [31].

The pre-hospital care time intervals can be a vital factor for improving management of pre-hospital systems. That often involves the time of emergency call; ambulance departure to scene; arrival at the scene; patients’ transportation and arrival at hospital to present the patient [23, 26, 32]. However, because the GKMA currently lacks any predefined EMS systems, there’s no recommended pre-hospital time intervals for responding to or transporting patients from crash scenes to the closest appropriate medical facilities [15, 26, 32]. In regard to this, prior studies showed the interval relating to the “golden hour” (first hour after the injury) to vary between the time of injury and presentation of the victims to medical facilities. In a citywide Kampala study, 36% of severely injured patients were reported to have arrived at the MNRH more than 1 h after they were injured without having received any pre-hospital care [15]. A similar study at the MNRH reported that only 27.5% of patients arrived within 1 h following their injury [32]. Thus, disparities in emergency time intervals and delays in responding to victims at the scene and getting them to hospitals can even exacerbate the consequences of the injuries suffered. It can also contribute needlessly to the death of victims while they are still at the scene or in-transit to hospital. One way to remedy this is to carefully assign particular road lanes for use by limited categories of vehicles such as ambulances to use to help facilitate their quick passage through congested traffic locations when they transport patients.

Nonetheless, the results from this investigation indicate that quite considerate attention has and is being directed towards enhancing the skills and competency of the EMS specialists; training more EMS personnel and increasing the number of ambulances for dispatch for emergencies, especially by the government affiliated EMS agencies [36, 37]. The UNAS which was established in 2015 under the Ministry of Health has taken the lead, with their ultimate goal being to offer pre-hospital care and emergency care services needed in Uganda [37]. Already the UNAS has launched a number of well-equipped fleets of ambulances with teams of trained responders to provide the first national 24 h pre-hospital services for emergencies around the KMA and surrounding areas [36]. That is augmented by some structural measures identified include setting-up known public call centers; training more EMS personnel; and facilitating ambulances and their personnel to be undertaken Indeed, these are positive steps forward but requires adequate funding, establishing EMS training centers with relevant curriculums to help to train competent EMS personnel as well as increasing the scope and the accessibility to ambulance services since in 2016, UNAS observed that despite the presence of ambulances countrywide, less than 7% of patients arrive at health facilities by ambulance [37].

The absence of formal EMS systems like in the GKMA necessitates innovative and low-cost solutions to be devised to meet the growing need for prehospital trauma care [33]. One way to achieve this is through coordination. To this matter, the present study has also observed the existence of coordination among different EMS agencies and stakeholders (MNRH, UPF, KCCA, CUFH-N or KMH, Uganda Red Cross, AAR-Uganda Healthcare Limited (formerly known as African Air Rescue), St. John’s Ambulance and others), which is championed at the present by the UNAS. This should be seen as a very positive response to the United Nations Road Safety Collaborations resolutions that shows Uganda is committed to implementing them [38]. This co-ordination and collaboration also helps to mount multi-pronged response for victims during mass causality RTIs given that at times their response can overwhelm the capacities of any one responder. Indeed, a cost-effectiveness injury control strategy enhanced for the GKMA and Uganda as a whole is vital where there’s still substantial deficiency in EMS resources. Apart from that, good coordination also facilitates better pre-hospital emergency care preparedness and swift transportation which follows certain procedures by the ambulance crews as also identified in this study in order to ensure the safe transportation of victims to medical facilities. However, both effective coordination and victims’ transportation should be inclusive enough to involve a wide range of stakeholders in road safety and injury prevention including laypeople that often arrive at the crash site first before any other responders [25, 29, 32].

A potential limitation in this study is the collinearity to generalize different variables into a single weakness or capacity as a factor affecting the pre-hospital emergency care for victims of RTIs. This might have caused a loss of information with the contribution of some variables being either under or over estimated. Like any other cross-sectional studies, our study might have been susceptible to recall bias encountered when the respondents were answering the questions in the questionnaires. Also, the criteria for selecting variables for PCA was not well defined and at times the PCs could be selected randomly and subjectively based on the personal choice rather than any specific reason or systematic approach which could lead to some sources of bias. Additional limitation comes from the missing data for some cases although its percentages were not high. Nonetheless, the study findings based on the PCA, which is the first of its kind in Uganda, can and should be used to help to inform the injury control and prevention measures taken to respond to RTI victims in the GKMA.

## Conclusion

Notwithstanding some progress made, the factors identified to affect the pre-hospital emergency care for victims of RTIs in the GKMA basically emanate from the absence of predefined and organized EMS systems in Uganda. The findings herein should be useful for devising, establishing and improving the preventive and post-crash management measures which are undertaken by different road safety and injury prevention stakeholders in the quest to reduce preventable deaths, injuries morbidity, disabilities and economic losses caused by RTIs in the GKMA. Aside the injuries, any ongoing EMS initiatives should be comprehensive to cover a wide spectrum of communicable infections, non-communicable conditions and obstetrics, and be supported also with more research in the GKMA in particular and Uganda in general.

## Additional file

Additional file 1: A questionnaire intended to identify the capacities and weaknesses of emergency medical Systems for the victims of road traffic incidents in the Greater Kampala Metropolitan Area. (DOCX 412 kb)

## Abbreviations

AAR: Uganda Healthcare Limited
CUFH-N: China- Uganda Friendship Hospital Naguru
DALYs: Disability Adjusted Life Years lost
EMS: Emergency Medical Sciences
GDP: Gross Domestic Product
GKMA: Greater Kampala Metropolitan Area
HICs: High Income Countries
KMA: Kampala Metropolitan Area
KMH: Kibuli Muslim Hospital
LMICs: Low and Middle-Income Countries
MNRH: Mulago National Referral Hospital
PCA: Principal Component Analysis
RTIs: Road Traffic Incidents
SPSS: Statistical Package for the Social Sciences
TUMS: Tehran University of Medical Sciences
UNAS: Uganda National Ambulance Service
UNCST: Uganda National Council of Science and Technology
WHO: World Health Organization
