# Supplementary material for: Weaknesses and capacities affecting the Prehospital emergency care for victims of road traffic incidents in the greater Kampala metropolitan area: a cross-sectional study
Source: BMC Emerg Med. 2017 Oct 3;17:29. doi: 10.1186/s12873-017-0137-2 (PMC5627469; doi:10.1186/s12873-017-0137-2)
Supplement: Additional file 1: — A questionnaire intended to identify the capacities and weaknesses of emergency medical Systems for the victims of road traffic incidents in the Greater Kampala Metropolitan Area. (DOCX 412 kb) [file 12873_2017_137_MOESM1_ESM.pdf]

A Questionnaire Intended to Identify the Capacities and Weaknesses of Emergency Medical Systems for Victims of Road Traffic Incidents in the Greater Kampala Metropolitan Area

Consent form

Research Title: Risk Mapping of Road Traffic Incidents in Greater Kampala Metropolitan Area for Planning of Emergency Medical Services

I am (or representing) Balikuddembe Kimuli Joseph from the School of Public Health, Departme nt of Disaster and Emergency Health, Tehran University of Medical Science.

I am (he is) conducting a study on Risk Mapping of Road Traffic Incidents in Greater Kampala Metropolitan Area for Planning of Emergency Medical Services

Study purpose

You are kindly requested to take part in this third phase of study whose purpose is mainly to; "*Id entify the Capacities and Weaknesses of Emergency Medical Systems for Victims of Road Traffic Incidents in the Greater Kampala Metropolitan Area".* Your feedback will be based on/ or used i n developing the final report/ or paper aimed at improving the emergency medical services syste m in Kampala Metropolitan Area in particular and Uganda in general. Afterwards, the report/ or paper shall be disseminated to all the concerned policy makers and stakeholders for appropriate a ctions.

Study procedure, recruitment of respondents and data collection

1. During the study you will be asked several questions and you are free not to answer to any que stion

2. Section **A** applies to all respondents, **B** only to victims involved in Road Traffic Incidents (RT Is) within the Greater Kampala Metropolitan Area (GKMA), **C** to experts on Emergency Medica l Service (EMS), and **D** applies to Emergency Medical Service key informants, policy makers an d analysts

3. Permission was first sought from the concerned authorities to involve you (their subjects) in this study

4. For the victim (s), the interviewer before beginning to the interview process need to find-out w hether he/ she was involved in the road traffic crashes within Greater Kampala Metropolitan Are a. If not the case, then should not proceed with the interview

6. The consent of the interviewee shall be acquired before the beginning of interview

7. An interview questionnaire designed in a semi-structure format with pre-identified questions shall be used in acquiring answers/ or opinions of respondents

8. The interview will be conducted both in English and Luganda (common languages used in Greater Kampala Metropolitan Area)

9. For victims of road traffic incidents who are unable to be interviewed, their caretakers can ans wer to the questions on their behalf

10. In some circumstances at the end of the interview the patient(s), a simple token of quick reco very shall be given to them

Benefits of the study

There is no immediate benefit but the results of this study will be communicated to the Emergenc y Medical Service providers and the policy makers to improve the planning of Emergency Medic al Service all over the country. There is no financial benefit in participating in this study.

Risk /Discomfort

There are no risks posed to you since you will only be required to respond to some questions.

Rights of participant

Participation in this study is voluntary and no penalty will be incurred for non-participation. You are free to leave any part(s) which you are not conversant with or not applicable to you. You also are free to withdraw from the study.

In case of any queries, please contact myself (Balikuddembe Kimuli Joseph) at 0772950519/075 2950519 OR Email: jbalikuddembe.k@gmail.com

For any question(s) regarding the clearance and approval of this research you may contact the Chair of Research and Ethics Committee - Mulago National Referral Hospital, Dr. Nakwagala Fred (0772-325869, Email: nakwagala@yahoo.com)

Confidentiality

Your identity will not be revealed in any presentations or publications of this study. The information obtained in the course of the study will only be communicated to no one else without your written explicit permission.

Statement of consent

I have been informed about the purpose of this study. I understand that questions will be asked of me. Participation in this study is entirely voluntary and refusal to participate will in no way affect my treatment. I am free to withdraw from the study. I therefore sign below as proof of my consent to participate in this study.

…………………………………………… ………………. ………………

Name of participant Signature/thumb print Date

…………………………...……… ………………. …… ..………………

Name of investigator/Research Assistant Signature/thumb print Date

………………………. ……………………….. ………………

Witness Signature/ thumb print Date

Questionnaire ID | | | | |

**A: Demographic information**		
A.1	Age	
A.2	Gender	☐ Male ☐ Female
A.3	Place of living	
A.4	Marital status	☐ Married ☐ Widow/Widower<br>☐ Not married ☐ Others (specify)
A.5	Nationality	☐ Ugandan ☐ Others (specify)
A.6	Employment/ occupation	
A.7	Organization/ Agency	
A.8	Job title/ position	
A.8	Level of education	☐ Diploma ☐ Primary School<br>☐ Bachelor's Degree ☐ Secondary School<br>☐ Master's Degree ☐ None<br>☐ Post Graduate ☐ Others (specify)
A.9	Years of work	☐ > 20 years ☐ 10 – 6 years<br>☐ 16 – 20 years ☐ 5 – 1 years<br>☐ 11 – 15Years ☐ < 1<br>☐ Have never worked
A.10	Any specialized training	
**B: Victims of Road Traffic Incident**		
B.1	Where did you get involved in the road traffic crash?	☐ Within the city center<br>☐ Other areas within GKMA (specify) …………………...
B.2	What was the mode of the road traffic crash?	☐ Private car ☐ Boda – boda<br>☐ Heavy omnibus (Bus) ☐ Heavy truck

		☐ Light omnibus (Taxi) ☐ Others …………
B.3	When did the road traffic crash happen?	☐ Morning hours ☐ Evening hours<br>☐ Afternoon hours ☐ Night hours
B.4	Who provided you with the initial care at the crash scene (if you recall, and were not unconscious)?	☐ Lay people ☐ Ambulance<br>☐ Police ☐ others (specify) ……………
B.5	How long did the responder reach you at the scene following the accident (please estimate the time if you can)?	☐ Within 5 - 10 Minutes ☐ Beyond 1 hour<br>☐ Within 30 - 1 Minutes ☐ Cannot recall
B.6	How were you transported from the crash scene to the hospital or any medical unit?	☐ Police vehicle ☐ Samaritan's car<br>☐ Ambulance ☐ Public transport<br>☐ Others (specify) ……………………….
B.7	Did you receive any treatment before reaching the hospital or any medical unit?	☐ Yes ☐ No<br>☐ Cannot recall
B.8	If Yes, what kind of treatment?	☐ Bandaging ☐ Fracture immobilization<br>☐ Wound dressing ☐ Airway management<br>☐ Others (specify) ……………………….
B.9	What kind of injuries did you sustain and your body parts that were affected following the crash?	☐ Head ☐ Abdominal and back<br>☐ Neck ☐ Limbs<br>☐ Chest ☐ Others (specify) ……………..
**C: Emergency Care Specialists, First Responders, Rescuers or Experts**		
C.1	Do you have a functional ambulance on standby?	☐ Yes ☐ No
C.2	How do you or your ambulance attendants receive notification about traffic accident?	☐ Two way radio signal ☐ Samaritan notification<br>☐ Toll free call ☐ Emergency call<br>☐ Others (specify) ……………………….
C.3	How does the ambulance dispatch team communicate with drivers?	☐ Two way radio signal ☐ Mobile phone<br>☐ Surveillance system ☐ Others (specify) ……

C.4	What kind of communication is used by the ambulance team communicate while transporting the victim?	☐ Two way radio signal ☐ Mobile phone<br>☐ Surveillance system ☐ Others (specify) ……
C.5	What type of ambulances do you use in transporting the victims of RTIs?	☐ Basic life support (BLS) ambulances<br>☐ Intermediate life support (ILS) ambulances<br>☐ Advanced life support (ALS) ambulances<br>☐ Others (specify) ……………………….
C.6	How many ambulances does your institution or company own?	☐ 1 - 3 ☐ 6 - 10<br>☐ 4 - 5 ☐ < 10
C.7	What are the qualification requirements of the ambulatory team?	☐ BLS Certificate ☐ Degree Certificate<br>☐ ALS Certificate ☐ Others (specify ………
C.8	How are your ambulances facilitated in terms of communication equipment, personnel and medication?	☐ EMT personnel and BLS equipment<br>☐ EMT personnel and ALS equipment<br>☐ EMT personnel, ALS, BLS and communication equipment<br>☐ Others (specify)
C.9	Is there any coordination between you and other key emergency responders during emergency response?	☐ Yes (Specify)<br>☐ No
C.10	Is there any special advice to ambulance attendants when en route victims?	☐ Yes (Specify)<br>☐ No
C.11	What do you base on when deciding where to take victims of crash?	☐ Severity of injuries ☐ Patient's wish<br>☐ Services of recipient hospital ☐ Distance<br>☐ Others (specify) ………………………………..
C.12	Are there any radio channels or telephone contacts that are held specifically for accidents or any other emergencies?	☐ Yes (Specify)<br>☐ No
C.13	Do you conduct triage in case of road traffic incidents involving mass causality victims?	☐ Yes<br>☐ No
C.14	How is the victims' transportation effectively done in order ensure fastest movement of ambulance or rescue	☐ Use siren and alarm to clear road<br>☐ Use police escort vehicles

	vehicle from accident scene to hospital or any other medical unit?	☐ Maneuver through diversion roads<br>☐ Others (specify) ………………………………..
C.15	Who pays for the ambulance services offered?	☐ Victim ☐ Government<br>☐ Insurance ☐ Charity<br>☐ Others (specify) ………………………………..
C.16	What kind of medical services are offered to the patient while being transported in ambulance?	☐ Basic Life Support<br>☐ Advanced Life Support<br>☐ Others (specify) ………………………………..
C.17	How many daily calls do you receive and respond to?	☐ < 5 ☐ 10 - 20<br>☐ 5 – 10 ☐ > 20
C.18	Is there specific period of time; area; or days when you receive increased calls for response to road traffic crashes?	☐ Yes (Specify)<br>☐ No
C.19	How many cases of road traffic crashes do your ambulance services attend to per day or per week?	☐ < 5 ☐ 10 - 20<br>☐ 5 – 10 ☐ > 20
C.20	What are the most common cases of road traffic accidents you get called to respond to?	☐ Private car ☐ 2/3 wheelers<br>☐ Heavy omnibus (bus) ☐ Heavy truck<br>☐ Light omnibus (taxi) ☐ Others (specify)
C.21	Do you provide regular training to your emergency responders or ambulance attendants and how often?	☐ Yes (Specify)<br>☐ No
C.22	Is there any procedure taken to bypass the receiving hospitals or unit(s) where victim(s) are transported first in case do not have necessary response services?	☐ Yes (Specify)<br>☐ No
C.23	What is the biggest challenge you face in ensuring effective pre-hospital EMS?	☐ Inappropriate equipment ☐ Poor roads<br>☐ Insufficient training ☐ Traffic jam<br>☐ Funding ☐ Patient handing over<br>☐ Others (specify)……………

Thanking you!
